# Biomechanical differences between two different shapes of oblique lumbar interbody fusion cages on whether to add posterior internal fixation system: a finite element analysis

**DOI:** 10.1186/s13018-023-04461-6

**Published:** 2023-12-13

**Authors:** Jianchao Liu, Ziming Geng, Jian Wang, Zepei Zhang, Xingze Zhang, Jun Miao

**Affiliations:** Department of Spine Surgery, Tianjin Hospital, Tianjin University, No. 406 Jiefang South Rd, Hexi District, Tianjin, 300211 China

**Keywords:** Oblique lumbar interbody fusion, Biomechanical evaluation, Finite element analysis, Cage subsidence, Cage subsidence

## Abstract

**Background:**

Oblique lateral lumbar fusion (OLIF) is widely used in spinal degeneration, deformity and other diseases. The purpose of this study was to investigate the biomechanical differences between two different shapes of OLIF cages on whether to add posterior internal fixation system, using finite element analysis.

**Methods:**

A complete three-dimensional finite element model is established and verified for L3–L5. Surgical simulation was performed on the verified model, and the L4–L5 was the surgical segment. A total of the stand-alone group (Model A1, Model B1) and the BPSF group (Model A2, Model B2) were constructed. The four OLIF surgical models were: A1. Stand-alone OLIF with a kidney-shaped Cage; B1. Stand-alone OLIF with a straight cage; A2. OLIF with a kidney-shaped cage + BPSF; B2. Stand-alone OLIF with a straight cage + BPSF, respectively. The differences in the range of motion of the surgical segment (ROM), equivalent stress peak of the cage (ESPC), the maximum equivalent stress of the endplate (MESE) and the maximum stress of the internal fixation (MSIF) were compared between different models.

**Results:**

All OLIF surgical models showed that ROM declines between 74.87 and 96.77% at L4–L5 operative levels. The decreasing order of ROM was Model A2 > Model B2 > Model A1 > Model A2. In addition, the ESPC and MESE of Model A2 are smaller than those of other OLIF models. Except for the left-bending position, the MSIF of Model B2 increased by 1.51–16.69% compared with Model A2 in each position. The maximum value of MESE was 124.4 Mpa for Model B1 in the backward extension position, and the minimum value was 7.91 Mpa for Model A2 in the right rotation. Stand-alone group showed significantly higher ROMs and ESPCs than the BPSF group, with maximum values of 66.66% and 70.59%. For MESE, the BPSF group model can be reduced by 89.88% compared to the stand-alone group model.

**Conclusions:**

Compared with the traditional straight OLIF cage, the kidney-shaped OLIF cage can further improve the stability of the surgical segment, reduce ESPC, MESE and MSIF, and help to reduce the risk of cage subsidence.

## Introduction

Oblique lateral interbody fusion (OLIF), as a new minimally invasive spinal surgery technique proposed in recent years, has been widely used worldwide. The operation enters through the physiological gap between the retroperitoneal abdominal vascular sheath and the anterior edge of the psoas major muscle. An instrumental channel is inserted to treat the operative segmental disk, and a larger interbody fusion apparatus is inserted to open the intervertebral space to achieve indirect decompression of the spinal canal and foramina [1]. As a modified lateral approach technique, OLIF has been widely used for lumbar degenerative diseases, scoliosis, and infectious diseases of the lumbar spine. Compared to extreme lateral interbody fusion(XLIF) and direct lateral interbody fusion (DLIF), it effectively avoids injury to the psoas major muscle and lumbar plexus nerve because it does not pass through the psoas major muscle [2]. In addition, OLIF surgery does not require intraoperative neurophysiological monitoring, and the incidence of hip flexion weakness and thigh numbness is lower than that of XLIF and DLIF. Compared with posterior lumbar interbody fusion (PLIF), OLIF does not destroy the posterior structure of the lumbar spine and has the advantages of less trauma, less bleeding, lower probability of nerve injury, and faster postoperative recovery [3]. When it comes to treating single-segment degenerative lumbar spondylolisthesis, OLIF offers the following advantages: less surgical invasion, better decompression, and quicker postoperative recovery than transforaminal interbody fusion (TLIF) [4]. Reduced risk of abdominal macrovascular and abdominal organ damage is compared to anterior lumbar interbody fusion (ALIF) and advantages in the range of segmental applications, restoration of disk height and segmental lumbar lordosis [5]. It is now increasingly favored by surgeons. However, postoperative cage subsidence (CS) is the most common complication of OLIF, with an incidence of 10.1–46.7% [6–8]. Higher levels of CS result in loss of intervertebral height, as well as lower fusion rates and poorer clinical improvement, or even recurrence of symptoms and deterioration of neurological function, increasing the patient's cost and medical burden [9]. To reduce the occurrence of CS, it is critical that a good adjunctive internal fixation scheme is selected during the operation accompanied by an appropriate interbody fusion cage [10]. As far as we know, there have been many studies on cage subsidence after OLIF operation in recent years. Current biomechanical concerns have focused on the combination of OLIF with different posterior assisted fixation systems, while cage itself does not seem to have received much attention [11–14]. In addition, some previous biomechanical experiments on OLIF used traditional straight interbody fusion cage, and few people studied the biomechanical differences of cage with different shapes after OLIF surgery. Based on the above reasons, according to the Chinese unique intervertebral height parameters and the radial design of vertebral endplate, we cooperated with professional engineers to design a kidney-shaped cage in SolidWorks software and finally produced it in Fule (Beijing, China). Three-dimensional finite element analysis (FEA) was used to analyze the biomechanical properties of the OLIF cage to determine whether it can reduce the risk of postoperative CS, assist the surgeon during surgery in selecting the appropriate interbody fusion cage, and provide clinical advice.

FEA, as a computational technique, primarily employs mathematical approximations to simulate real-world physical scenarios. Initially utilized for structural strength calculations in aerospace, it has progressively gained widespread use in recent years within the field of medical orthopedic engineering, owing to the ubiquitous advancement and rapid evolution of computer technologies. It is playing an increasingly pivotal role in this domain [15]. FEA can accurately predict the response of a novel implant under various loads, simulate the mechanical state of the implant inside the human body, and visualize stress and deformation quantitatively and intuitively. It effectively reflects the characteristics of the implant. With the ongoing software updates, FEA methods can precisely simulate different surgical scenarios, allowing for the artificial setup of experimental conditions and multiple repetitions of experiments. As a robust alternative to cadaveric and animal experiments, it offers advantages such as easy operation, convenient data acquisition, reliable experimental results, and significant time and cost efficiency. The application of finite element analysis enables simulated testing of newly designed cages before their manufacture, serving as a crucial complementary approach to clinical and in vitro experiments [16].

## Materials and methods

### Establishment of L3–L5 lumbar spine model

**Fig. 1:**
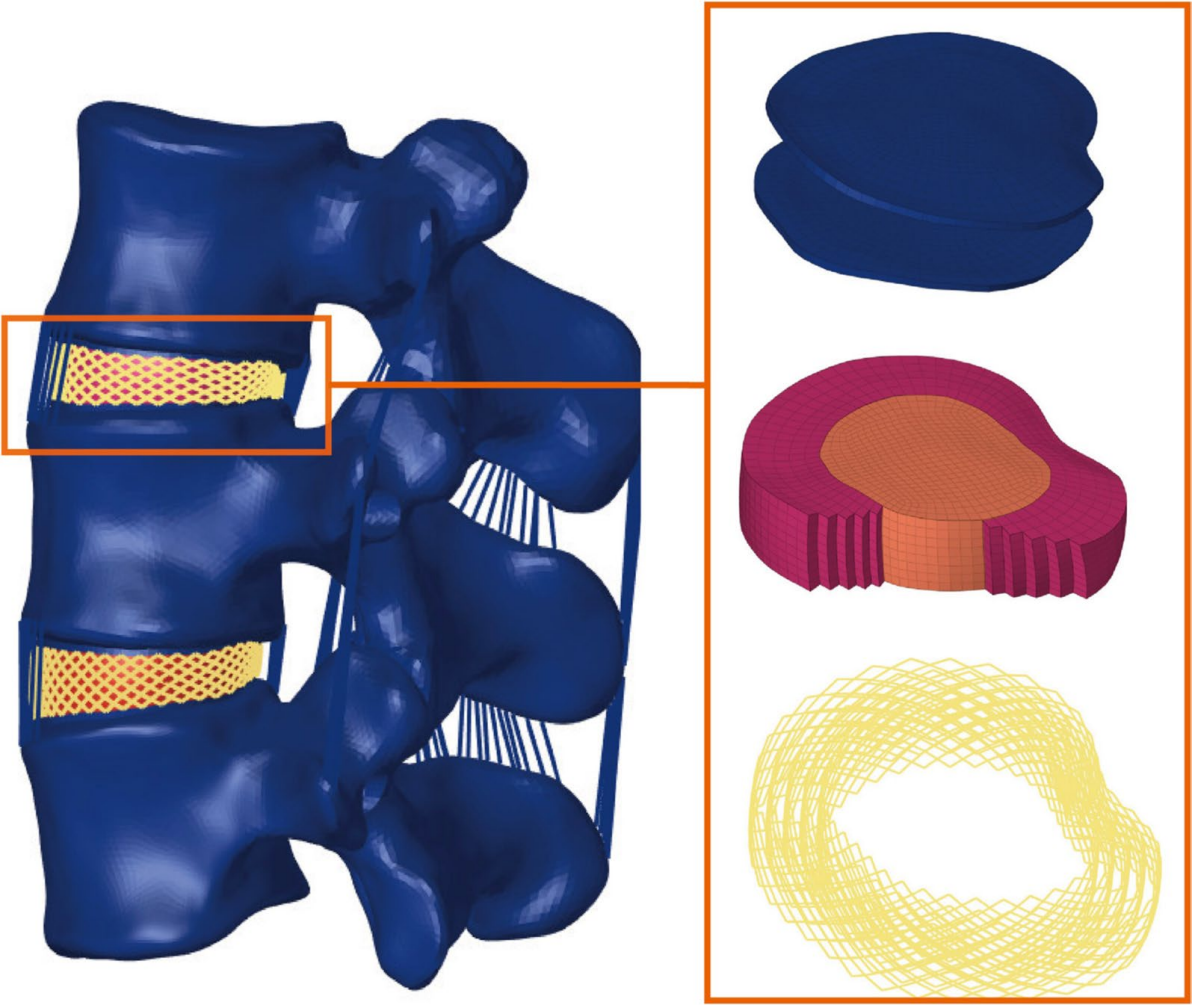
The complete L3–L5 model

We selected one healthy volunteer (male, 170 cm height, 70 kg weight) without any history of lumbar deformities, tumors, traumas, surgeries, or other diseases. By X-ray imaging examination, exclude spinal fracture, deformity, bone destruction and other lesions. The volunteers were informed about the experiment and signed relevant informed consent after being approved by the Ethics committee of Tianjin University Tianjin Hospital. Sixty-four slice spiral CT (Siemens, Germany) was used to perform continuous thin-layer scanning (layer thickness 0.625 mm) on the lumbar vertebrae of volunteer men, and DICOM format was derived. Using commercial software Mimics 20.0 (materialize, Leuven, Belgium) read the data and extracted the L3–5 model. After a simple repair, it was poured into Geomagic Studio v12.0 (Geomagic, Research Triangle Park, NC, USA) to repair the model, fit the surface, and construct the cortical bone and cancellous bone model. The model was imported into SolidWorks 2021 (Dassault Systemes, Paris, France) in step format to construct the lumbar posterior structure, facet joint, nucleus pulposus, annulus fibrosus, and endplate structure. The processed model is imported into Hypermesh 2019 ( Altair Engineering, Troy, MI, USA) in step format to mesh and construct structures such as ligaments. The model is assembled, material attributes are assigned, set up contact, stress analyses, and test mesh convergence in Abaqus 2020 (Simulia, Johnston, RI, USA). In this study, the complete model for L3–L5 included cortical bone, cancellous bone, posterior structure, articular cartilage, endplate, intervertebral disk and ligaments, the thickness of articular cartilage was 2 mm, and the interaction between articular surfaces was face-to-face contact. The friction coefficient was 0.1. The thickness of cortical bone was 1 mm. The thickness of upper and lower endplates was 0.5 mm [17, 18]. The intervertebral disk is composed of the nucleus pulposus, stroma and annulus fibers, which are embedded into the annulus matrix in the form of truss units. The nucleus pulposus comprises approximately 46% of the disk volume, and the annulus stroma comprises approximately 54% of the disk volume. The annulus fibers contain multiple layers and are angled approximately ± 30° from the endplate surface [19]. The ligaments were simulated according to the corresponding anatomical structures, including anterior longitudinal ligament, posterior longitudinal ligament, ligamentum flavum, interspinous ligament, supraspinous ligament, joint capsule ligament, and intertransverse ligament. Both ligament and annulus fibrosus fibers are simulated by truss element (T3D2) and only subjected to tensile load. Figure 1 shows the complete L3–L5 model.The material properties of each component are based on previous studies (Table 1) [20–22].

**Table 1:** Material properties used by finite element model

Component	Young’s modulus (MPa)	Poisson’s ratio	Cross-sectional area (mm^2^)
*Vertebra*			
Cortical bone	12,000	0.3	
Cancellous bone	100	0.2	
Posterior element	3500	0.25	
Sacrum	5000	0.2	
Facet	11	0.2	
*Disk*			
Endplate	24	0.4	
Nucleus pulpous	1	0.49	
Annulus ground substance stance	4	0.4	
Annulus fibers	360–550		0.15
*Ligaments*			
ALL	7.8		63.7
PLL	10		20
LF	15		40
CL	7.5		30
ISL	10		40
SSL	8		30
ITL	10		1.58
*Implants*			
Cage	3600		
Screws and roots	110,000		

*AL*L anterior longitudinal ligament; *PLL* posterior longitudinal ligament; *LF* ligamentum flavum; *CL* capsule ligament; *ISL* interspinous ligament; *SSL* supraspinous ligament; *ITL* intertransverse ligament

### Development of the surgical lumbar spine model

**Fig. 2:**
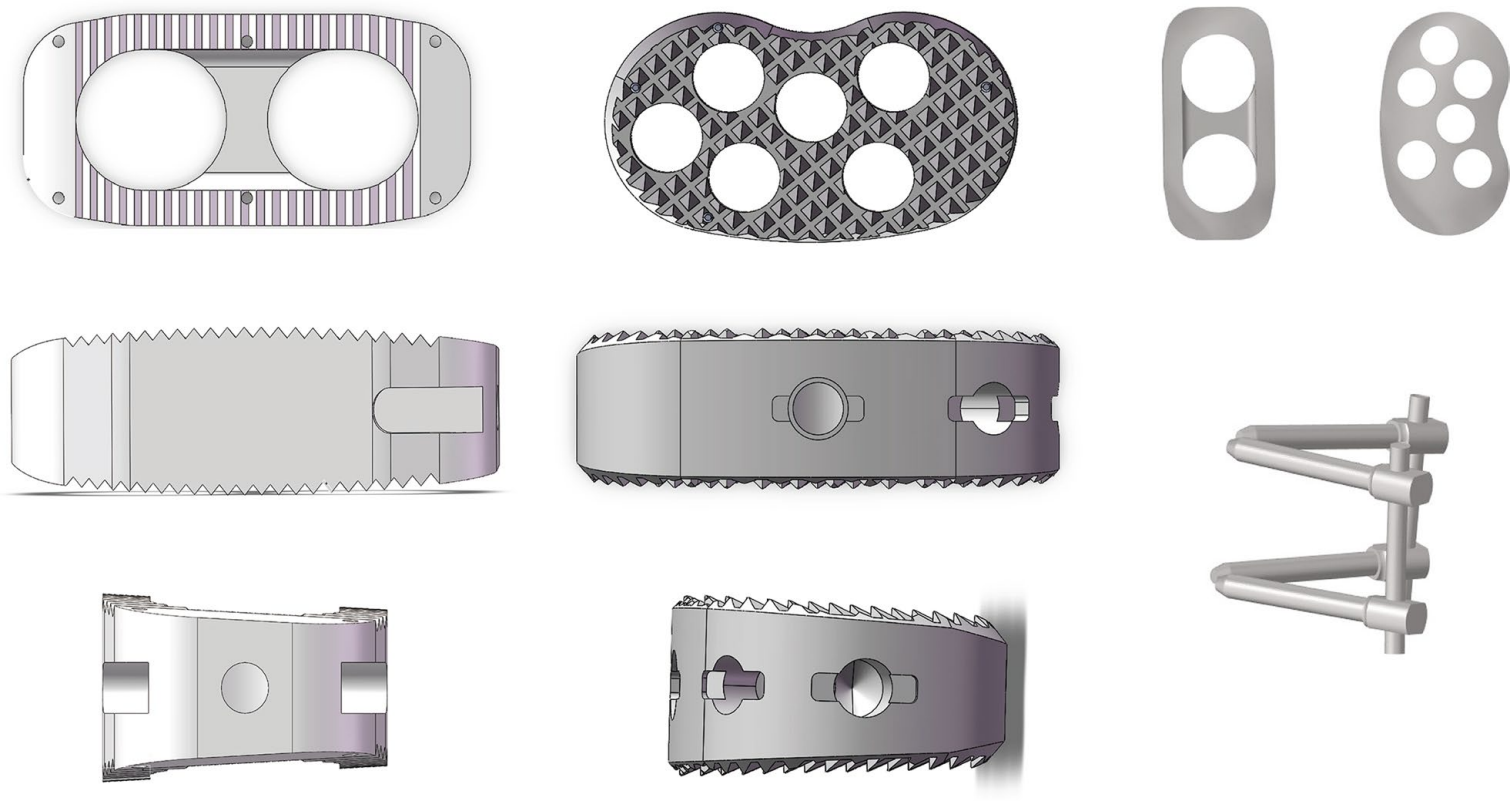
Kidney-shaped cage, straight cage, bilateral pedicle screws fixation

**Fig. 3:**
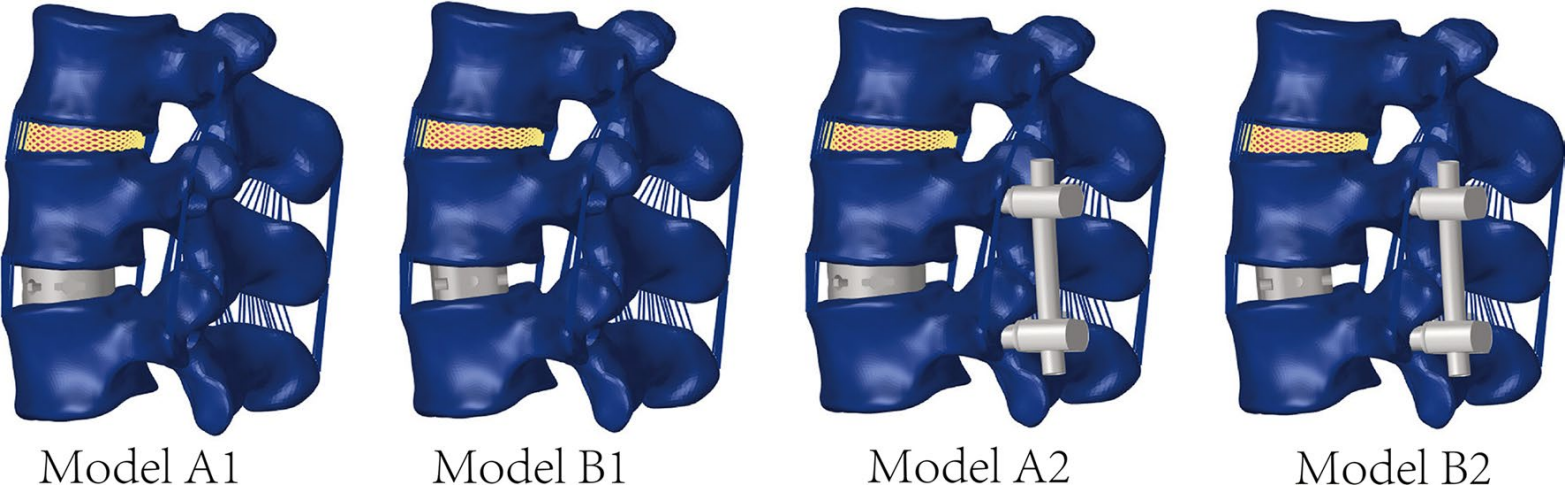
Four OLIF surgical models: **A1**. Stand-alone OLIF with a kidney-shaped Cage, B1. Stand-alone OLIF with a straight Cage, **A2**. OLIF with a kidney-shaped Cage + BPSF, **B2**. Stand-alone OLIF with a straight Cage + BPSF

Considering that L4–L5 is a common segment of lumbar degeneration, L4–L5 is selected as the surgical segment. The intervertebral disk and cartilage endplate were removed at this segment, and the intervertebral fusion cage was placed on the left side, ensure that cage completely spans the epiphyseal ring. The intervertebral fusion cage was modeled based on physical objects in SolidWorks 2021 (Dassault Systemes, Paris, France). The size of the straight cage is 40 × 18 × 11 mm, the convex angle is 6°, and the size of kidney-shaped cage was 40 × 22 × 11 mm, and the convex angle is 6° (Fig. 2).The simplified pedicle screw has a length of 50 mm and a diameter of 6.5 mm, and the diameter of the connecting rod is 5.5 mm (Fig. 2). In order to simplify the OLIF procedure, the serrations on the surface of the interbody fusion cage were removed and the overlapped parts of the endplate were removed using Boolean operation to achieve a geometric match between the upper and lower endplates and the cage (Fig. 2) [23]. Finally, we constructed the stand-alone group ( Model A1, Model B1) and BPSF group (Model A2, Model B2) models by deleting the relevant repeated grids in Hypermesh software. The four OLIF surgical models were: A1. stand-alone OLIF with a kidney-shaped cage; B1. stand-alone OLIF with a straight cage; A2. OLIF with a kidney-shaped cage + BPSF; B2. stand-alone OLIF with a straight cage + BPSF (Fig. 3). In the model, except for the contact setting between the lumbar facet joints ( as mentioned above), the rest of the contact settings are “bonded”.

### Loading and boundary conditions

In the ABAQUS software, boundary conditions and loads were set to establish appropriate constraints. Initially, all nodal degrees of freedom on the lower surface of the L5 vertebra were constrained to ensure rigid fixation in all degrees of freedom. Subsequently, a coupling node was established on the upper surface of the L3 vertebra to simulate a physiological compression load of 400 N, representing the gravitational force exerted by the body on the spine. Placing the mechanical loading at the coupling node allowed for an even distribution of the mechanical load on the surface of the L3 vertebra, rendering the mechanical loading more realistic. Simultaneously, a 7.5 N mm torque load was applied to further simulate the postures of the lumbar spine during flexion, extension, lateral bending, and axial rotation movements of the human body.

### Evaluation indicators

In this study, we used the following four indicators to evaluate biomechanical properties: 1. the range of motion of the surgical segment (ROM), 2. equivalent stress peak of the cage (ESPC), 3. the maximum equivalent stress value of the endplate (MESE), 4. the equivalent stress value of the pedicle screw fixation system (MSIF).

## Results

### Validation of the model

**Fig. 4:**
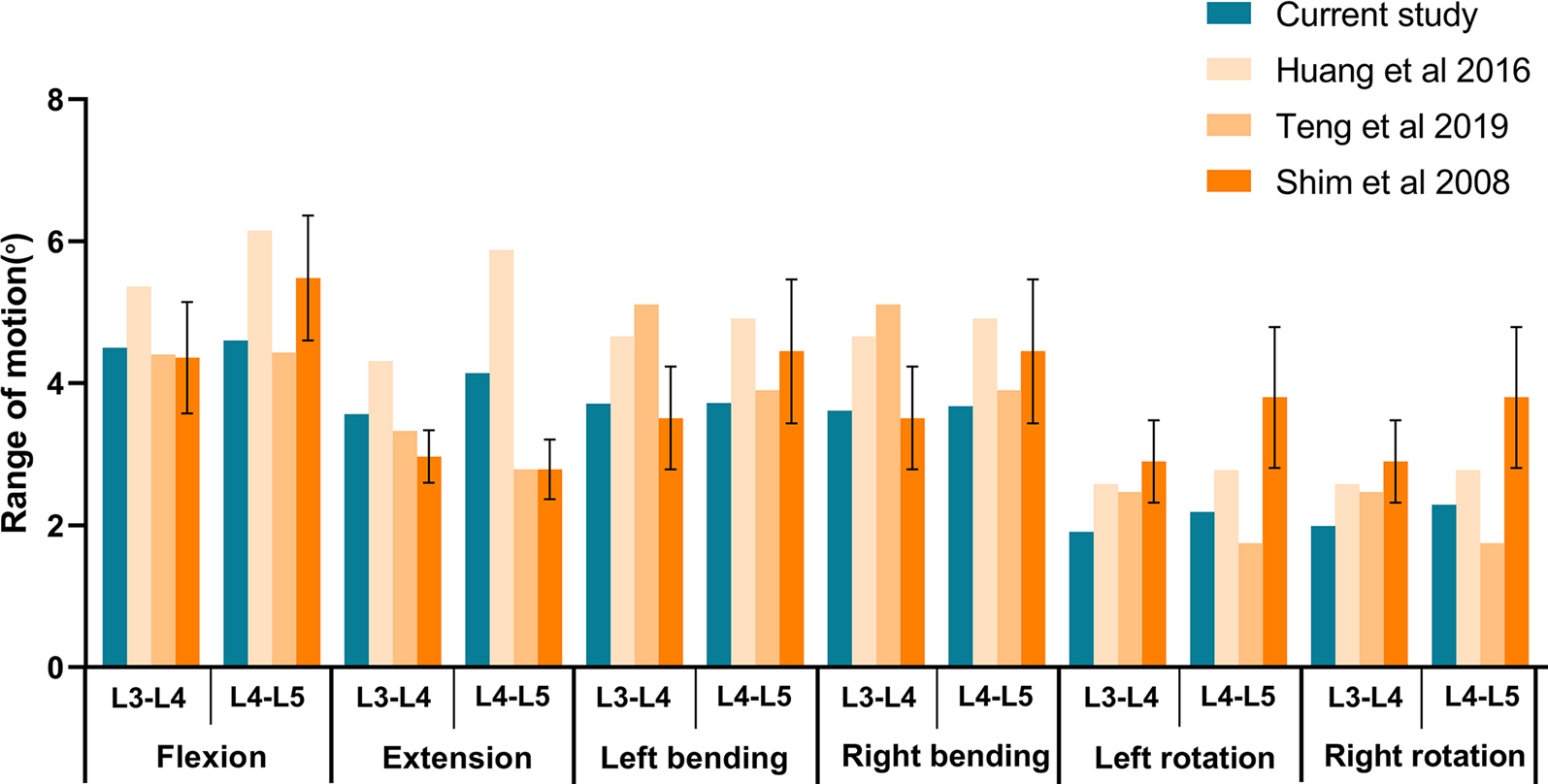
Our study was compared with other studies on the ROM in each lumbar segment

The complete model was subjected to the same load conditions as in previous literature. The range of motion (ROM) for each segment was measured under different motion states and compared with previous reports from cadaveric studies and finite element experiments (Fig. 4) [24–26]. Due to individual differences, there are also differences in lumbar segmental activity. The segmental activity of the model is within a reasonable range compared with the previous reports. Therefore, this study successfully constructed the L3–L5 finite element model, which can be used for further research.

### The range of motion of the surgical segment (ROM)

**Fig. 5:**
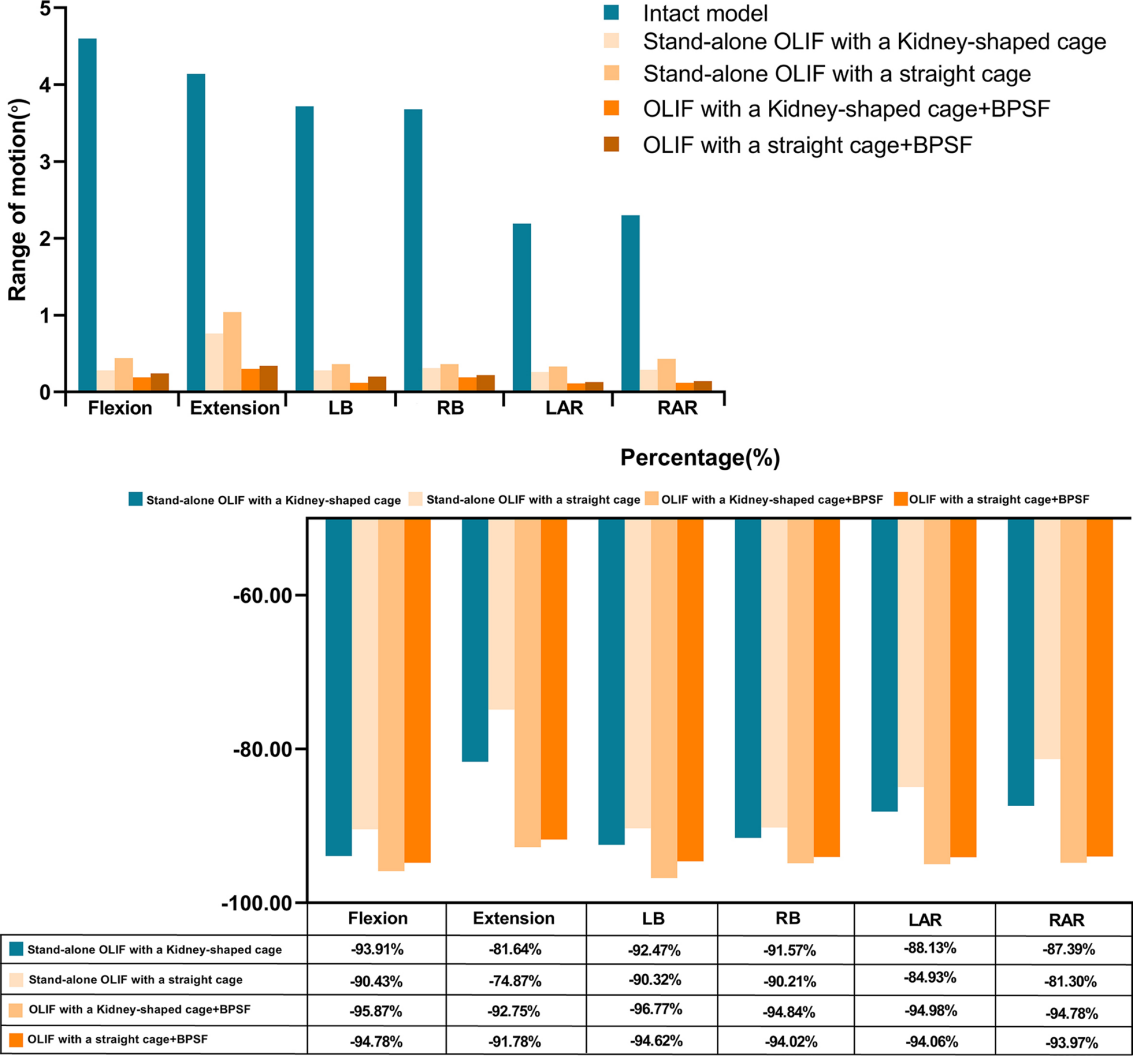
Comparison of ROM between segmental and surgical models of L4–L5

The ROM of L4–L5 of each model is shown in Fig. 5. Compared with the Intact model, the ROM of the two groups of models was significantly reduced.The largest reduction in ROM is model A2, the range of motion decreased by 95.87%, 92.75%, 96.77%, 94.84%, 94.98% and 94.78%, respectively, in flexion, extension, left-bending, right-bending, left-rotation and right-rotation. Model B1 had the least decrease in activity in all directions: ROM decreased by 90.43%, 74.87%, 90.32%, 90.21%, 84.93% and 81.3%, respectively, in all directions. The ROM of all models decreased in the order of A2 > B2 > A1 > A2. Compared with model A1, the ROM of model A2 increased by 16.12% to 57.14% in each position. The ROM of model B2 increased by 13.33% to 66.66% compared with model B1.

### Equivalent stress peak of the cage (ESPC)

**Fig. 6:**
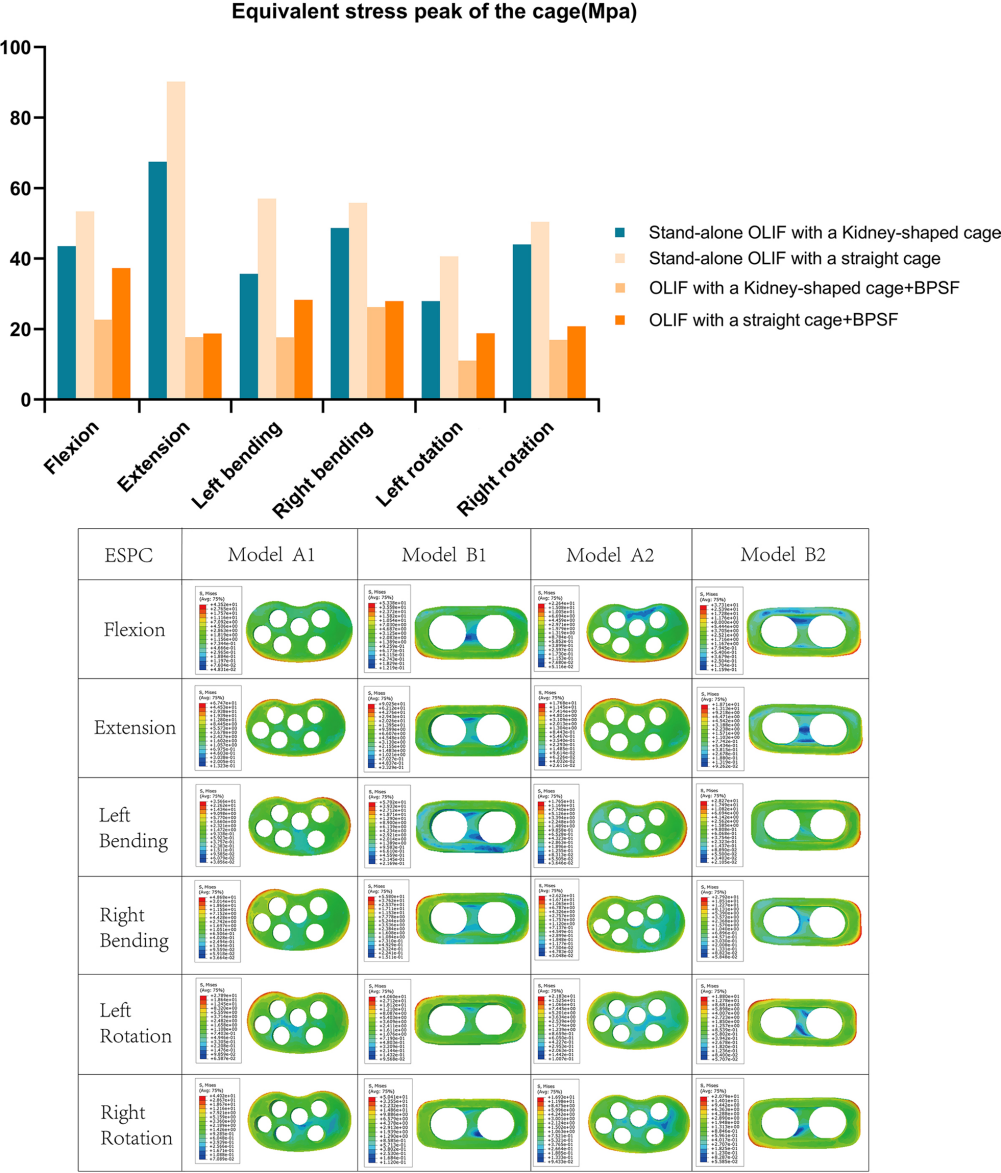
Equivalent stress peak of the cage in each surgical model

Figure 6 is the ESPC in different positions of each group of models. The maximum ESPC is model B1, and the ESPC in each position is 40.6–90.25 MPa. The minimum value of ESPC is model A2, from 11.02 to 26.22 Mpa. The two groups of ESPC from high to low are Model B1 > Model A1 > Model B2 > Model A2. The Model A1 ESPC was 27.89–67.47 Mpa in each position, and the model B2 ESPC was 18.71–37.31 Mpa in each position. Both models A1 and B1 were in the stand-alone OLIF group, compared with kidney-shaped cage, ESPC of straight cage increased by 14.51–59.89% in each position. Both models A2 and B2 were in the OLIF + BPSF group; the ESPC of the straight cage in model B2 was increased by 5.82–70.59% compared with that of the kidney-shaped cage in model A2.

### The maximum stress of the internal fixation (MSIF)

**Fig. 7:**
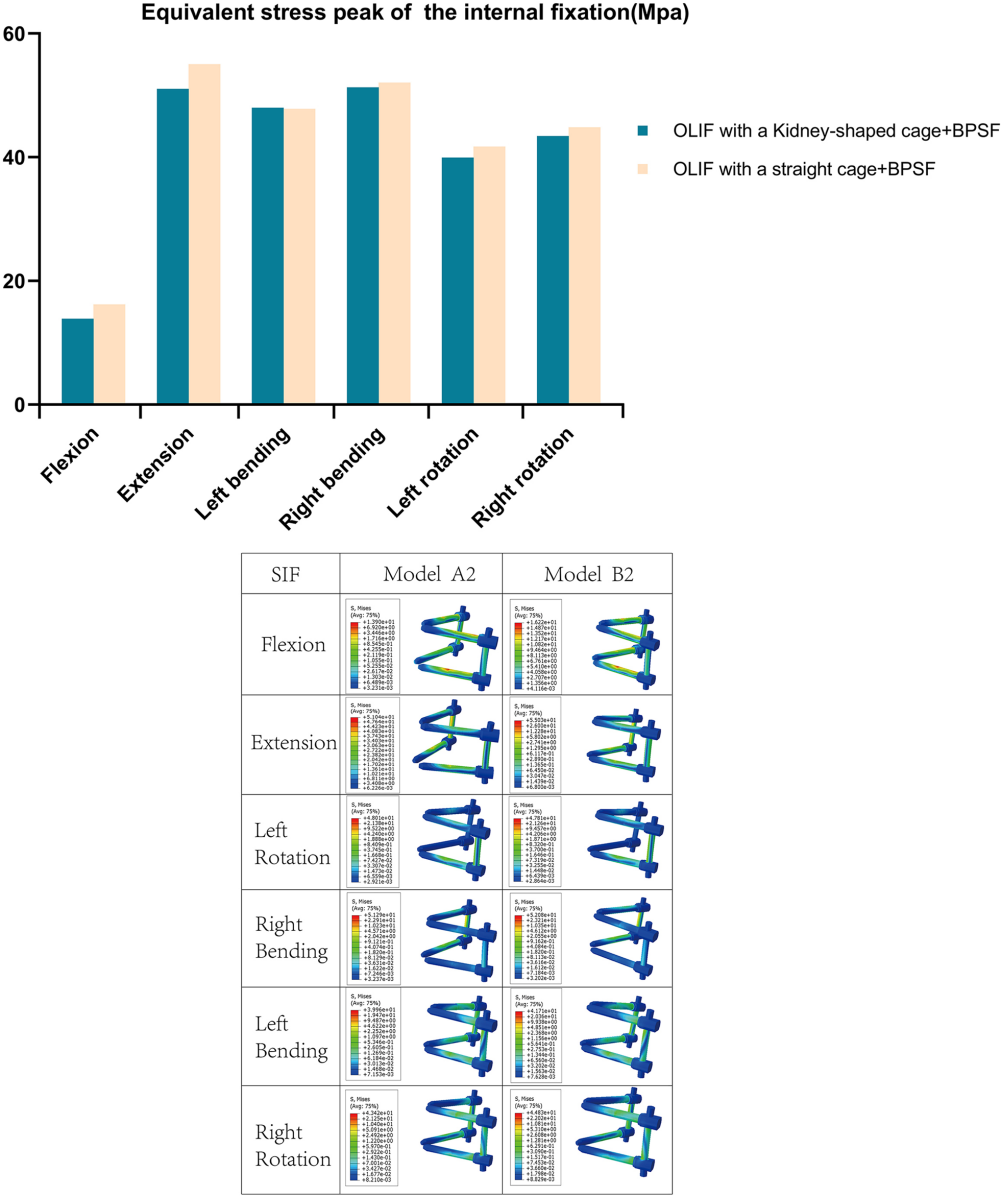
Von Miss stress of posterior pedicle screw fixation system

Figure 7 shows the MSIF and the stress distribution of model A2 and model B2. Except for the left bending (B2 was 47.81 Mpa, A2 was 48.01 Mpa), the MMSIF of model B2 in other positions increased by 16.69%, 7.81%, 1.51%, 4.37% and 3.24%, respectively, compared with that of A2 in flexion, extension, right bending, left-rotation and right-rotation positions.

### Maximum equivalent stress of endplate (MESE)

**Fig. 8:**
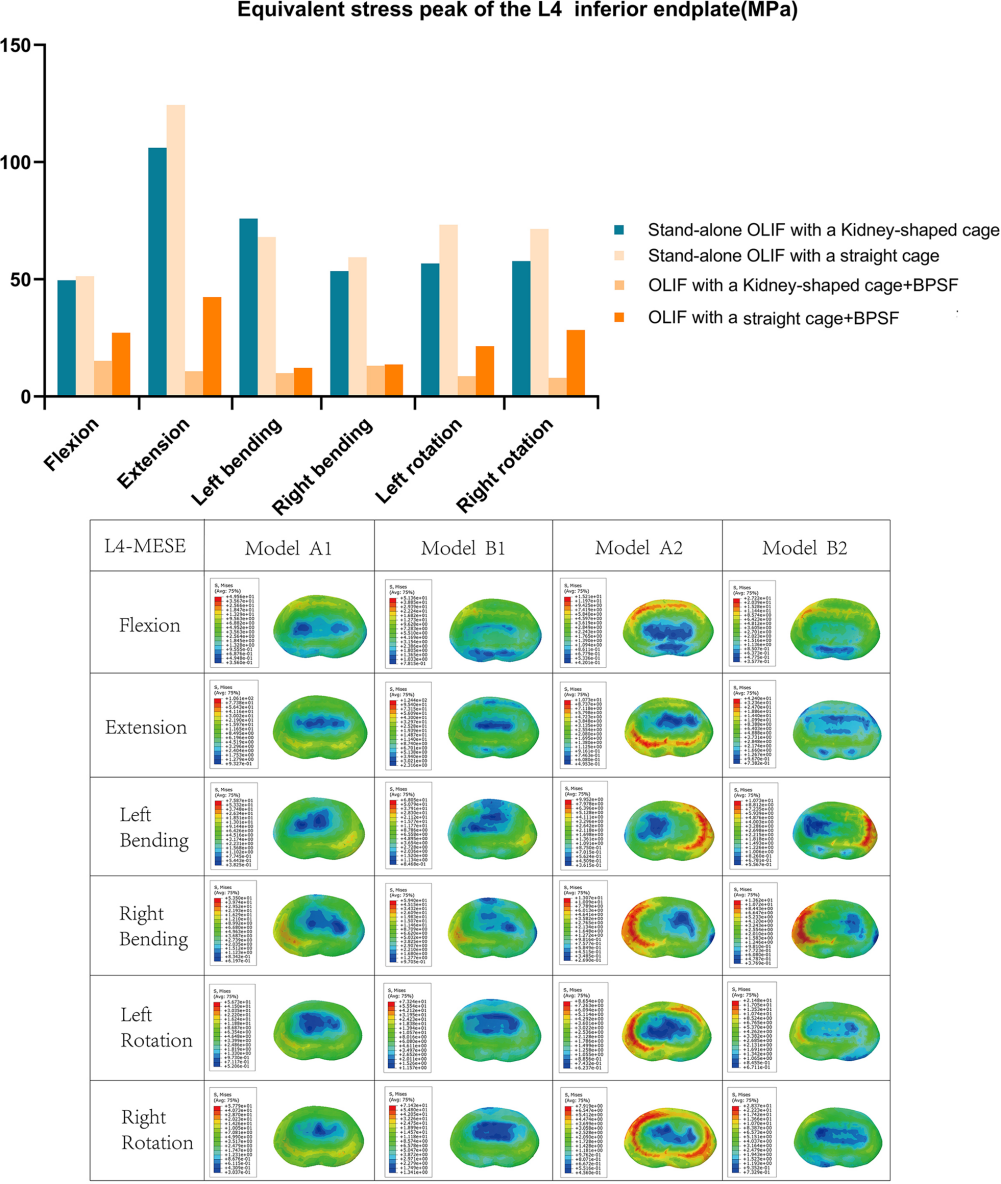
Stress in the L4 subendplate at each position for all models

**Fig. 9:**
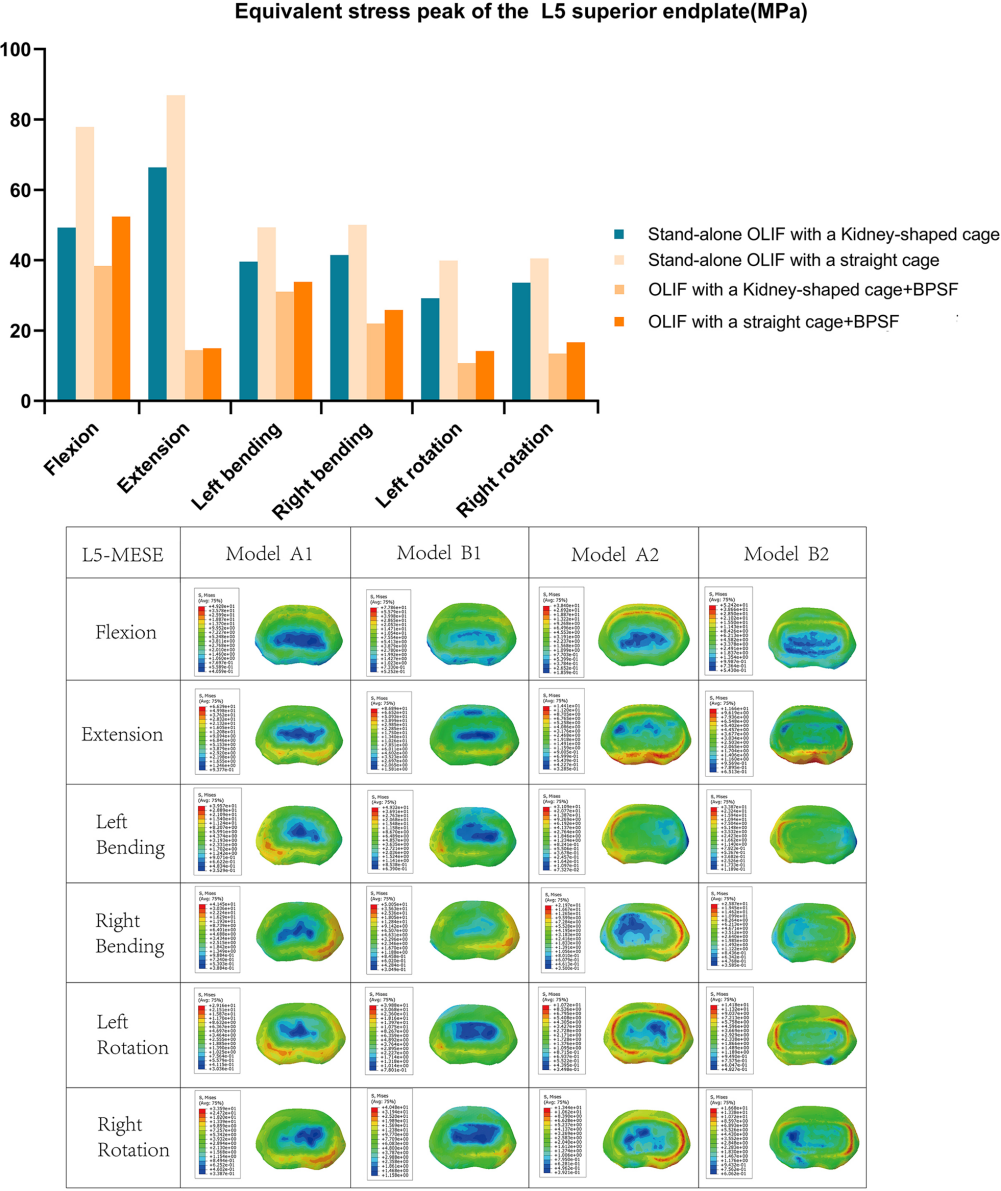
Stress in the L5 superior endplate at each position for all models

Figures 8 and 9 show the stress distribution of l5 upper endplate and L4 lower endplate; the stress is mostly concentrated around the contact area between the endplate and the cage. For the L4 lower endplate, the minimum MESE is model A2, and MESE is 7.91–15.21 Mpa in each position. Except for L4 lower endplate MESE at left bending (Model A1 is 75.87 Mpa, Model B1 is 68.05 Mpa). The maximum MESE in each position was model B1, The MESE of L4 lower endplate and L5 upper endplate is 59.4–124.4 Mpa, 39.88–86.89 Mpa. For model A2 and model B2 of OLIF + BPSF group, MESE was less than that of stand-alone group. The MESE of L4 upper endplate and L5 lower endplate of model A1 and model B2 in each position were 53.5–106.1 Mpa and 29.16–66.39 Mpa, 12.19–42.4 Mpa, 14.18–52.42 Mpa, respectively. In the L4 lower endplate MESE, except for the left-bending position, the model B1 increased by 3.63%, 17.24%, 11.02%, 29.10% and 23.60%, respectively, compared with the model A1. Compared with A1 and B1, the MESE of L4 lower endplate and L5 upper endplate decreased by 47–89.88% and 21.43–82.73% in model A2 and model B2, respectively.

## Discussion

In the past two decades, minimally invasive spine surgery has made significant progress, attracting great interest from surgeons and patients [27]. OLIF was first proposed by Silvestre in 2012 and has been increasingly used by spinal surgeons, and research on OLIF is also growing rapidly [28, 29]. Endplate injury and cage subsidence after OLIF are important predictors of postoperative revision, which have also plagued surgeons and patients and cannot be ignored. According to previous studies, the incidence of cage-related complications after OLIF was 2.9% to 13.4% [30]. Zhao et al. [9] found that 79 (32.6%) of 242 patients with OLIF were diagnosed with CS. Kotheeranurak et al. [8] found that CS occurred in 50 (46.7%) of 107 patients undergoing OLIF. Prevention of postoperative complications and selection of appropriate cage play an important role in the postoperative effect. From the biomechanical point of view, the greater the contact area between the cage and the endplate, the more effective the dispersion of the load stress between the cage and the endplate. This can reduce the occurrence of stress concentration, thereby reducing postoperative endplate damage. In the biomechanical test, the wider cage selected during the operation will obtain a larger cage and endplate contact area, and better segmental stability can be obtained after the operation [31]. The traditional straight cage commonly used in OLIF surgery is generally 18 mm wide, Marchi et al. [32] found that at 1-year follow-up, 30% lumbar cage with a width of 18 mm had high-grade CS after stand-alone lateral interbody fusion, while only 11% lumbar cage with a width of 22 mm had CS. Cai et al. [33] designed five different shapes of cervical cage (square, oval, kidney-shaped, clover-shaped, and 12-leaf-shaped) and evaluated the biomechanical properties by finite element method. It was found that the kidney-shaped cervical fusion cage had good biomechanical properties and was the best choice for fusion segments.

Based on this, we designed a lumbar kidney-shaped OLIF cage with a length of 40 mm, a width of 22 mm, and a height of 11 mm, 13 mm, and 15 mm. In this study, FEA was used to evaluate the biomechanical differences between kidney-shaped and straight cage in OLIF. At present, there are a variety of OLIF surgical methods. As for the two mainstream surgical methods, stand-alone OLIF and OLIF + BPSF, a lot of clinical follow-up work has been done. Compared with OLIF + BPSF, stand-alone OLIF can significantly reduce the operation time, blood loss, operation cost and avoid lumbar and back muscle injury, and can significantly improve the early clinical effect after operation was no significant difference in long-term clinical and radiological results [29, 34]. Zeng et al. [35] followed up 235 patients undergoing OLIF, of which 22 patients had endplate injury and 18 patients had cage subsidence and lateral displacement. The incidence of CS in stand-alone OLIF group was higher than that in OLIF + BPSF group. There are many reasons for endplate injury and cage subsidence after OLIF, such as obesity, osteoporosis, intraoperative iatrogenic endplate injury, intraoperative cage placement, and too small cage [35, 36]. Many scholars have found that OLIF + BPSF is an ideal internal fixation method and has the best biomechanical properties through the study of OLIF combined with different internal fixation systems [11, 37]. However, excessive rigid fixation with additional posterior pedicle screw fixation system also increases the risk of adjacent segment degeneration and vertebral instrument-related osteoporosis [38, 39].

At present, there is controversy over the choice of assisted internal fixation for OLIF surgery, and surgeons also choose different surgical methods based on different patients. Therefore, in this study, we designed two kinds of OLIF cage combined with stand-alone and BPSF, respectively, a total of four models, four evaluation indicators to evaluate the biomechanical properties of two different shapes of OLIF cage. The stability of the surgical segment is the key and goal of lumbar fusion. The greater the postoperative segment rigidity, the smaller the ROM, and the stronger the ability to limit displacement and deformation. Postoperative segmental instability can increase the incidence of complications, such as subsidence and non-fusion [11, 13]. Cai et al. [11] developed five OLIF surgical models, and the ROM of all surgical segments decreased by more than 80%. Among them, the ROM of the stand-alone OLIF group decreased by 86.26–94.51% in all motion directions. Our study is consistent with it. In our study, the stand-alone OLIF group reduced ROM by 74.87–93.91% in each position. In the stand-alone OLIF group, the ROM of combined kidney-shaped cage decreased more than that of combined straight cage in each position. This may be related to the kidney-shaped cage we designed. The kidney-shaped cage is closer to the shape of the physiological intervertebral disk. Compared with the straight cage, it has a larger effective contact area of cage-endplate, which is more advantageous in maintaining the stability of the spine. For the OLIF + BPSF group, the activity of each position can be reduced by 91.78–96.77%, which is also close to the results of Cai et al. [11]. In each position, the smallest ROM was the kidney-shaped cage + BPSF group. When OLIF combined with BPSF, we found that the kidney-shaped cage also has certain advantages for the stability of the fusion segment, but the difference of ROM between the two groups is very small. This also suggests that when OLIF combined with BPSF, the strong internal fixation effect of pedicle screw through three columns of vertebral body provides very good stability, and the advantages of cage 's own design are much smaller. From a biomechanical point of view, the success of long-term cage placement depends on effective load transfer, cage must apply sufficient mechanical stimulation to the endplate to promote bone formation and remodeling, while maintaining the bone–implant interface stress within a certain range to prevent implant subsidence or loosening. For MESE, the greater the stress, the more prone to CS. Our study showed that both stand-alone OLIF group and OLIF + BPSF group, kidney-shaped cage still has great biomechanical advantages over straight cage. The kidney-shaped cage has a greater ability to resist CS than the straight cage. With the delay of time, the risk of CS in the kidney-shaped cage and the reduction of intervertebral height is also reduced, the serious loss of intervertebral height may lead to the relaxation of ligament structure, that is, the so-called ' stretch-compression tension band effect ', which will lose the necessary stable biomechanical environment during spinal fusion and eventually lead to a decrease in fusion rate [40].

In this study, the minimum MESE was model A2 (kidney-shaped cage + BPSF), which was only 7.91 Mpa at right rotation, the maximum MESE is model B1 (Stand-alone straight cage), and the maximum stress can reach 124.4 Mpa at extension. The previous failure load for cortical bone was 90–200 Mpa [21]. In this study, the stress generated by model B1 during extension was already in this range, indicating that the risk of CS after surgery was greater when straight cage was applied alone in OLIF surgery. When OLIF was combined with BPSF, the posterior pedicle screw system significantly shared load conduction in the anterior and central columns of the vertebral body, thus significantly reducing the stress of the cortical bone endplate. From the perspective of ESPC and MESE, the kidney-shaped cage we invented has less stress at the bone–implant interface than the traditional straight OLIF cage, which indicates that it has great advantages in resisting CS risk. In addition, we also recorded the MSIF.In terms of MSIF, except for left-bending (Model B2 is 47.81 Mpa, Model A2 is 48.01 Mpa), model B2 increased by 1.51–16.69% compared with A2 in each position (In the left-bending position, the A2 model is 0.2 Mpa larger than the B2 model. We speculate that this may be related to the endplate of the vertebral body. The endplate of the volunteer is not absolutely flat, so this result is caused). This also indicates that the shape of the Cage has a certain effect on the posterior auxiliary internal fixation system, and the kidney-shaped cage reduces the stress on the posterior internal fixation system. This is significant as lower stress levels decrease the risk of bone destruction on the spine and screw contact surface, especially for patients with poor bone quality. Chen et al. [41] showed that the yield stress of titanium alloy was 897–1034 Mpa. The maximum equivalent stress of pedicle screw and rod in our model was much smaller than this value. Therefore, for this study, the risk of internal fixation failure in the two groups was very small.

To the best of our knowledge, this study, for the first time, utilizes FEA to evaluate the biomechanical differences between a novel kidney-shaped cage and the conventional rectangular cage with or without the addition of bilateral pedicle screw fixation systems. There are few clinical studies on different shapes of OLIF cage. The newly designed kidney-shaped OLIF cage has been applied clinically and is expected to be followed up in the future. Our results need to be further confirmed.

This study has some limitations. Firstly, we do not simulate the whole spine and paravertebral muscle and surrounding soft tissue and other complete human body model, cannot determine the impact of surrounding soft tissue and muscle on biomechanics. Secondly, the material properties we apply are determined based on the values given in the previous literature. These values are different from the actual human experimental values and cannot reflect the gap between individuals. Thirdly, the simple simulation of the skeleton structure is uniform and isotropic, while the skeleton itself is a complex heterogeneous and anisotropic composite material. Its response to the load is time-dependent [42]. Although there are some limitations, according to the FEA results, the kidney-shaped OLIF cage has great biomechanical advantages in resisting CS and endplate collapse compared with the straight OLIF cage, which has guiding significance for clinical surgeons.

## Conclusion

Our study showed that the kidney-shaped OLIF cage had better biomechanical properties than the traditional straight cage and that the intraoperative application of the kidney-shaped OLIF cage and the pedicle screw fixation system better protected against the risk of endplate injury and CS.

## Data Availability

Please contact the corresponding author for data requests.

## Abbreviations

FEA: Finite element analysis
OLIF: Oblique lateral lumbar fusion
PLIF: Posterior lumbar interbody fusion
XLIF: Extreme lateral interbody fusion
DLIF: Direct lateral interbody fusion
PLIF: Posterior lumbar interbody fusion
TLIF: Transforaminal interbody fusion
ALIF: Anterior lumbar interbody fusion
ROM: Range of motion
ESPC: Equivalent stress peak of the cage
MESE: Maximum equivalent stress of the endplate
MSIF: Maximum stress of the internal fixation
CS: Cage subsidence
